# Cardiac MRI-based right-to-left ventricular blood pool T2 relaxation times ratio correlates with exercise capacity in patients with chronic heart failure

**DOI:** 10.1186/s12968-023-00943-y

**Published:** 2023-06-19

**Authors:** Moritz C. Halfmann, Lukas Müller, Urs von Henning, Roman Kloeckner, Theresia Schöler, Karl-Friedrich Kreitner, Christoph Düber, Philip Wenzel, Akos Varga-Szemes, Sebastian Göbel, Tilman Emrich

**Affiliations:** 1 Department of Diagnostic and Interventional Radiology, University Medical Center of the Johannes Gutenberg-University Mainz, Langenbeckst. 1, 55131 Mainz, Germany; 2 German Center for Cardiovascular Research (DZHK), Partner Site Rhine-Main, Langenbeckst. 1, 55131 Mainz, Germany; 3 Department of Cardiology, University Medical Center Mainz-Center of Cardiology, Johannes Gutenberg University, Langenbeckst.1, 55131 Mainz, Germany; 4 Department for Interventional Radiology, University Hospital of Lübeck, Ratzeburger Allee 160, Lübeck, Germany; 5 Division of Cardiovascular Imaging, Department of Radiology and Radiological Science, Medical University of South Carolina, Ashley River Tower, 5 Courtenay Drive, Charleston, SC 29425-2260 USA; 6 Preventive Cardiology and Preventive Medicine, Center for Cardiology, University Medical Center of the Johannes Gutenberg-University Mainz, Langenbeckst. 1, 55131 Mainz, Germany

**Keywords:** Heart failure, T2 mapping, Exercise capacity

## Abstract

**Background:**

MRI T2 mapping has been proven to be sensitive to the level of blood oxygenation. We hypothesized that impaired exercise capacity in chronic heart failure is associated with a greater difference between right (RV) to left ventricular (LV) blood pool T2 relaxation times due to a higher level of peripheral blood desaturation, compared to patients with preserved exercise capacity and to healthy controls.

**Methods:**

Patients with chronic heart failure (n = 70) who had undergone both cardiac MRI (CMR) and a 6-min walk test (6MWT) were retrospectively identified. Propensity score matched healthy individuals (n = 35) served as control group. CMR analyses included cine acquisitions and T2 mapping to obtain blood pool T2 relaxation times of the RV and LV. Following common practice, age- and gender-adjusted nominal distances and respective percentiles were calculated for the 6MWT. The relationship between the RV/LV T2 blood pool ratio and the results from 6MWT were evaluated by Spearman’s correlation coefficients and regression analyses. Inter-group differences were assessed by independent t-tests and univariate analysis of variance.

**Results:**

The RV/LV T2 ratio moderately correlated with the percentiles of nominal distances in the 6MWT (r = 0.66) while ejection fraction, end-diastolic and end-systolic volumes showed no correlation (r = 0.09, 0.07 and − 0.01, respectively). In addition, there were significant differences in the RV/LV T2 ratio between patients with and without significant post-exercise dyspnea (p = 0.001). Regression analyses showed that RV/LV T2 ratio was an independent predictor of the distance walked and the presence of post-exercise dyspnea (p < 0.001).

**Conclusion:**

The proposed RV/LV T2 ratio, obtained by two simple measurements on a routinely acquired four-chamber T2 map, was superior to established parameters of cardiac function to predict exercise capacity and the presence of post-exercise dyspnea in patients with chronic heart failure.

**Supplementary Information:**

The online version contains supplementary material available at 10.1186/s12968-023-00943-y.

## Background

Heart failure (HF) is estimated to affect approximately 26 million people worldwide with prevalence steadily increasing due to an aging society [1–3]. Despite the overall improving prognosis, its 5-year mortality remains high and is characterized by poor quality of life [4]. Hence, there is a great scientific interest into HF, with the US National Library of Medicine listing over a thousand currently active studies on the topic. Besides typical endpoints including major adverse cardiac events (MACE), numerous surrogate parameters have been developed and are under constant re-evaluation.

One of those, the 6-min walking test (6MWT), a simple walking exercise, was first proposed in 1985 and since has been rigorously validated in clinical and scientific contexts [5–10]. The 6MWT has been shown to correlate with prognosis, therefore it got anchored in international HF guidelines and has evolved as the gold standard for the validation of new tests on functional capacity of patients [4, 11, 12].

Cardiac MRI (CMR) has a class I recommendation for the characterization of myocardial tissue and ventricular function according to the joint recommendations by the Society for Cardiovascular Magnetic Resonance (SCMR) and the European Association for Cardiovascular Imaging (EACVI) as well as the guidelines by the European Society of Cardiology (ESC) [12, 13].

In addition, the current clinical recommendations by the SCMR state that parametric mapping provides incremental value in the workup of patients with heart failure [13]. Specifically, T2 mapping allows for voxel-wise quantification of relaxation times and is primarily used to estimate myocardial oedema [14]. When this technique is applied to the blood pool, paramagnetic effects of deoxygenized haemoglobin compared to diamagnetic oxygenated haemoglobin can lead to a dephasing of spins within the erythrocytes [15, 16]. This in turn causes the shift of water protons by means of diffusion along local field gradients and thus results in a correlation of blood pool T2 relaxation times with levels of blood oxygenation [15–18].

Based on this mechanism, we hypothesized that impaired exercise capacity in HF is associated with a greater difference between right (RV) to left ventricular (LV) blood pool T2 relaxation times due to a higher level of peripheral blood desaturation, compared to patients with preserved exercise capacity and to healthy controls (HC). Thus, the aim of this study was to correlate the RV/LV T2 ratio with functional exercise capacity in patients with chronic HF who had previously undergone both CMR and 6MWT.

## Methods

### Study population

The local ethics committee approved the protocol of this retrospective study with a waiver for informed consent [reference number 837.196.13 (8881-F)].

A total of 271 patients who underwent CMR in 2021 were screened for the following inclusion criteria: (1) presence of chronic HF according to current guidelines [12]; (2) complete CMR including T2 mapping without severe artefacts; and (3) successfully performed 6MWT. After survey, 142 patients were excluded for the absence of HF, lung disease as the primary cause of symptoms or for being bed-ridden and therefore unable to complete the 6MWT.

**Fig. 1:**
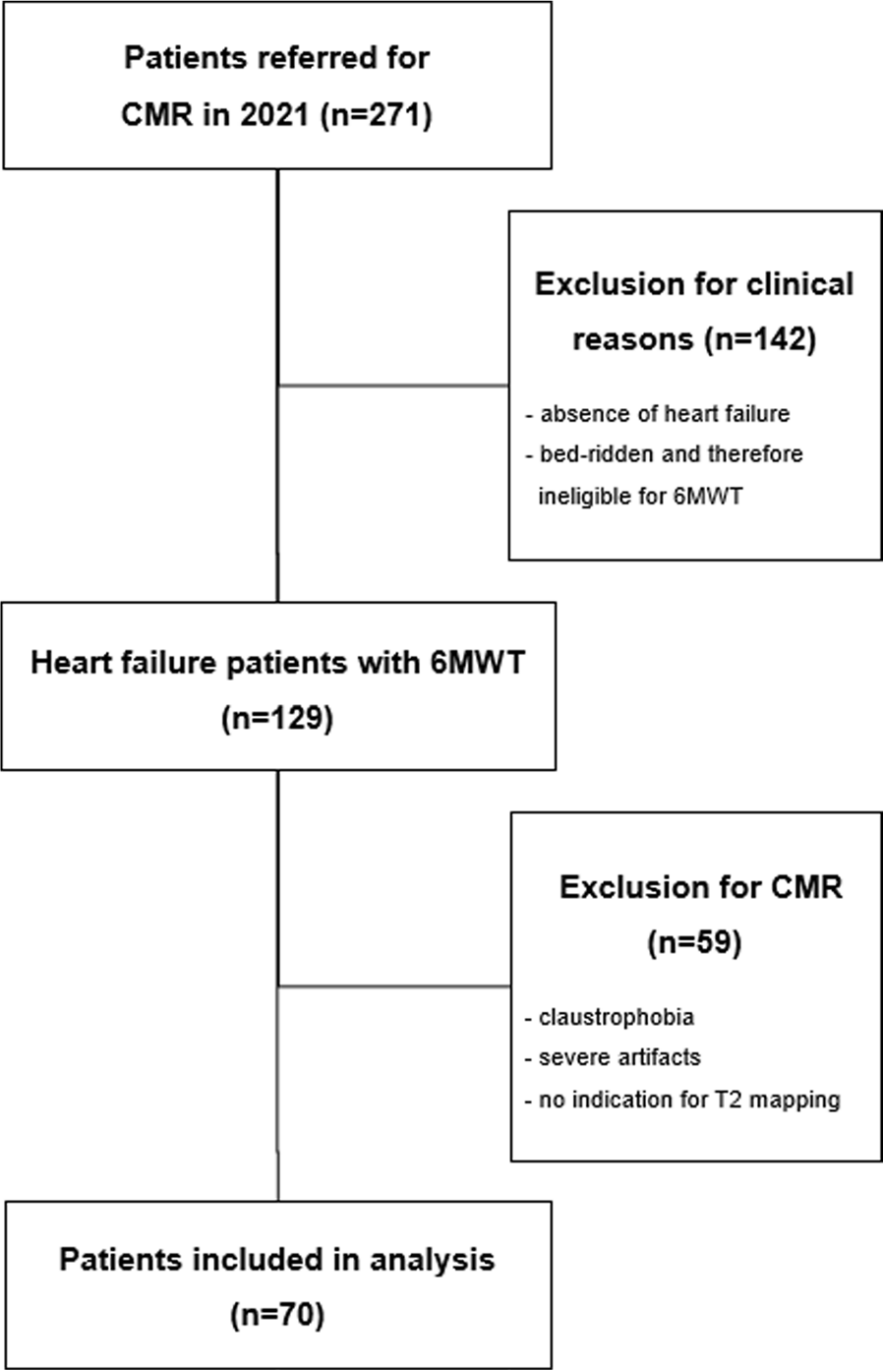
Study flowchart

Image quality assessment was performed by 2 independent observers (T.E. and M.C.H.) with 13 and 5 years of experience in the field, respectively. A third observer (A.V.S., 15 years of experience) mediated disagreement. The quality assessment was conducted based on the protocol validated by Klinke et al*.* within the European CMR registry [19]. Image slice thickness (8 mm) and inter-slice gap (2 mm) as well as the angulation of the image stacks were defined by institutional standard operating procedures. Failure to adhere to these in-house guidelines or incomplete LV-coverage resulted in the exclusion of the patient study. In addition, shortened study protocols without T2 mapping (i.e. due to limited breath-hold capabilities of the patient) were also excluded from the study. The remaining studies were evaluated for the presence and extent of the following artifacts: wrap around, respiratory/cardiac ghosting, image blurring/mis-triggering, metallic artifacts, shimming artifacts and signal loss. In total, this led to the exclusion of an additional 59 patients due to incomplete or insufficient quality of CMR, resulting in a total of 70 patients who were included in the study (Fig. 1).

Additionally, 35 propensity score matched HC, who had previously participated in a prospective study to establish institutional CMR reference values, were included in this study. Propensity score matching was performed based on factors that are known to influence 6MWT including age, sex, weight, and height.

Demographic parameters, results from the 6MWT, objective lung function analysis results, and HF symptoms as rated on the New York Heart Association (NYHA) scale were derived from the electronic patient records.

### Cardiac magnetic resonance imaging

All subjects underwent CMR (Software version XA30, Magnetom Prisma®, Siemens Healthineers, Erlangen, Germany) at 3 Tesla (T). Short- and long-axis views of standard balanced steady-state free precession cine acquisitions, T1 and T2 maps in short-axis views and four-chamber views were included among other routine sequences in a comprehensive clinical protocol. For T2 mapping, a commercially available sequence with 3 preparation pulses (0/30/55 ms) and a 3-heartbeat recovery period was used. All maps were acquired in the diastolic phase. Additional pulse sequence parameters can be found in Table 1.

**Table 1:** Key CMR pulse sequence parameters for cine imaging and T2 mapping

	Cine	T2 mapping
Sequence type	bSSFP cine	T2 prepared bSSFP
ECG gating	Retrospective	Retrospective
Repetition time (ms)	3.88	3.15
Echo time (ms)	1.42	1.32
Field of view (mm)	360	360
Phases/cardiac cycle	25	n/a
Slice thickness (mm)	8.0	8.0
Flip angle (°)	60	12
Voxel size (mm^3^)	1.5 × 1.5 × 8.0	1.9 × 1.9 × 8.0
Bandwidth (Hz/pixel)	930	1185

*bSSFP* balanced steady-state free precession, *ECG* electrocardiogram

End-diastolic (EDV) and end-systolic (ESV) volumes, ejection fraction (EF), and cardiac output index (CI) were calculated for both ventricles by semi-automatically drawn endocardial contours on the short-axis stack, using all three long-axis views as references. Subsequently, all volumes were indexed to body surface area (BSA).

**Fig. 2:**
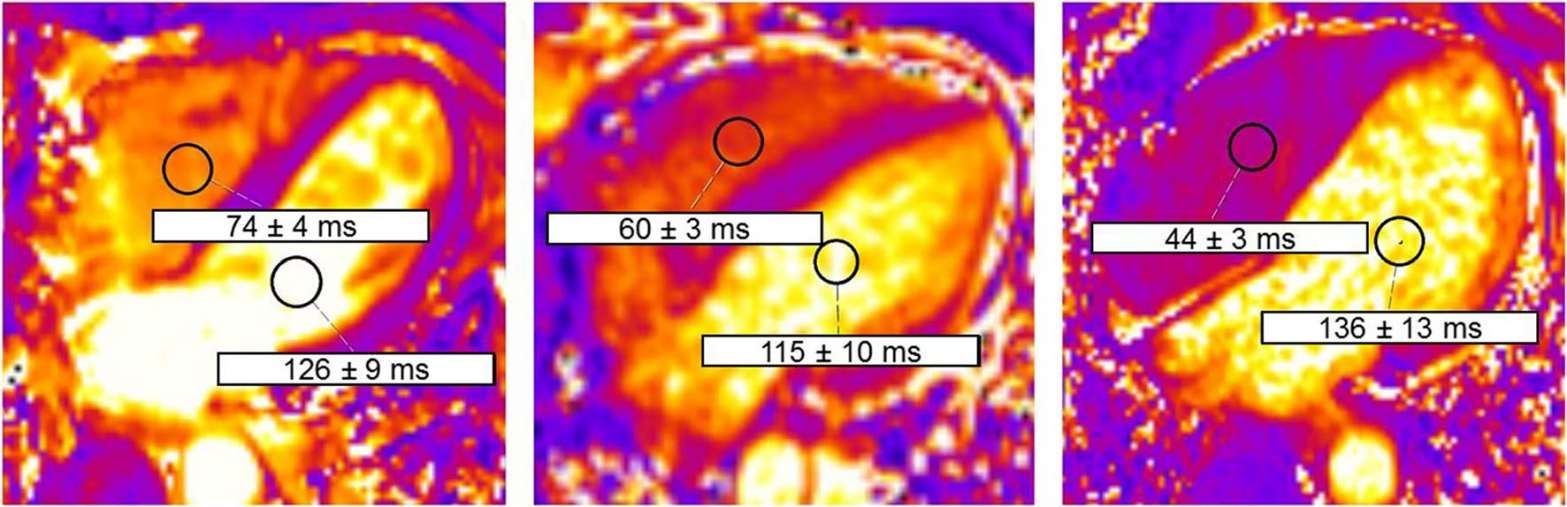
ROI placement in the RV and LV blood pool in T2 maps of a 62-year-old healthy man (left), a 67-year-old woman with HF (middle), and a 66-year-old man with HF (right). *ROI* region of interest, *RV* right ventricle, *LV* left ventricle, *HF* heart failure

For T2 mapping analysis, the source data was carefully reviewed to exclude flow artefacts. Subsequently, two circular regions of interest (ROI) with a minimum size of 1.5 cm^2^ were placed in artefact free areas of the RV and LV blood pool, and the mean relaxation times were recorded (Fig. 2).

Midventricular short-axis slices of native and post-contrast maps were semi-automatically segmented with a 25% endo- and epicardial offset margin. Together with haematocrit values, these were then used to calculate extracellular volume fraction (ECV). Quantitative assessment of late gadolinium enhancement (LGE) was performed using the semi-automated 5-standard deviation (SD) method on basal, midventricular and apical short-axis slices on phase-sensitive inversion recovery (PSIR) sequences acquired 10 min after administration of 0.2 mmol/kg gadoteric acid, as recommended by the SCMR [20]. Using the quantified amount of LGE, the myocardial scar burden was calculated as a percentage of myocardial mass.

### 6-min walking test

A trained nurse performed the 6MWT according to international guidelines [21, 22]. Prior to walking, the patient’s weight and height were noted. In addition, baseline systolic blood pressure (RR_sys_), heart rate (HR) and peripheral capillary oxygen saturation (SpO_2_) were measured. Patients were then instructed to walk as fast as possible on a marked course without changes of direction for six minutes. Changes in speed and breaks were allowed. After six minutes, the distance reached was recorded and post-exercise parameters were measured (RR_sys_, HR, and SpO_2_). In addition, the patient was asked to subjectively rate the level of dyspnoea on a modified Borg scale from 0 to 10. Patients with a subjective rating of 4 (“somewhat severe”) or higher were considered as having significant post-exercise dyspnoea in further analysis. As common practice, age and gender adjusted nominal distances and respective percentiles were calculated using the established formula by Troosters et al. [23].

### Statistical analysis

Statistical analysis was performed using SPSS version 23 (IBM Corporation, Armonk, New York, USA). All data were tested for normal distribution using the Kolmogorov–Smirnov test and were subsequently either reported as mean ± standard deviation or median with an interquartile range. Categorical data were reported as absolute frequencies and proportions.

Comparisons between groups and sub-groups were evaluated using independent t-tests or univariate analysis of variance (ANOVA), where appropriate. Correlations between distances and the RV/LV T2 ratio were assessed using Spearman's correlation coefficient. Univariate and multivariate linear regressions with block enter method and respective hazard-ratios were used to investigate predictors of the distance walked in the 6MWT. For multivariate regression analysis, variance inflation factors were calculated to test for multicollinearity. Factors of 5 or lower were considered acceptable. In addition, a binary logistic regression analysis was used to determine predictors for post exercise dyspnoea. In this retrospective study, statistical power for both the comparisons between cohorts and sub-cohorts as well as the regression models was assessed using a dedicated freeware tool (G*Power v3.1, https://www.psychologie.hhu.de/arbeitsgruppen/allgemeine-psychologie-und-arbeitspsychologie/gpower [24]). A p-value < 0.05 was considered significant.

For reproducibility analysis, intra- and inter-reader agreement were assessed by intraclass correlation coefficients. In addition, agreement between different methods of ROI placement was evaluated by Bland–Altman analysis and coefficient of variance determination.

## Results

### Study participants

The mean age in the patient cohort was 60 ± 15 years with 18 (26%) women while in HC the mean age was 55 ± 11 years with 14 (40%) women. Electronic patient records revealed that 39 (56%) of patients had never smoked, 17 (24%) were past-smokers and 14 (20%) were actively smoking. Despite matching efforts, the HF group had significantly higher body mass index (BMI) (27.4 ± 5.7 vs. 24.6 ± 4.7 kg/m^2^, p = 0.01).

### Cardiac magnetic resonance imaging

During image quality assessment, the observers were in disagreement in a total of 3 cases (observer 1: 59 exclusions, observer 2: 60 exclusions). Together with the mediation observer, a consensus was reached resulting in the following distribution of reasons for exclusion: Failure to adhere to in-house guidelines or incomplete LV-coverage (n = 7; 12% of excluded studies), shortened study protocol without T2 mapping (n = 36; 61%), wrap around artifacts (n = 1, 2%), respiratory/cardiac ghosting (n = 2, 3%), image blurring/mis-triggering (n = 8, 14%) metallic artifacts (n = 2, 3%), shimming artifacts/signal loss (“off-resonance”; n = 3, 5%).

Biventricular functional CMR analysis showed significant differences between HF patients and HC for all parameters except for CI (3.1 ± 1.3 vs. 3.0 ± 0.7, p = 0.994) and RVEDVi (93.4 ± 30.7 vs. 87.5 ± 15.5, p = 0.192). Additionally, the patient population was stratified by aetiology of HF as well as the categories of HF with reduced ejection fraction (HFrEF), HF with mildly reduced ejection fraction (HFmrEF) and HF with preserved ejection fraction (HFpEF). This information, along further baseline characteristics, can be found in Table 2. None of the CMR parameters differed significantly between patients referred for CMR in an acute setting such as first onset of HF symptoms (n = 28/70, 40%) and those undergoing a scheduled follow-up (n = 42/70, 60%) (all p > 0.100, Additional file 1: Table S1).

**Table 2:** Baseline characteristics

	Patients with HFrEF (n = 40)	Patients with HFmrEF (n = 8)	Patients with HFpEF (n = 22)	Healthy controls (n = 35)
Demographics				
Females (%)	11 (28)	0 (0)	7 (32)	14 (40)
Age (years ± SD)	63 ± 13	59 ± 11	56 ± 17	55 ± 11
BMI (kg/m^2^ ± SD)	27.0 ± 6.0	23.3 ± 2.5	29.7 ± 5.2	24.6 ± 4.7
BSA (m^2^ ± SD)	1.94 ± 0.27	1.89 ± 0.18	2.03 ± 0.18	1.89 ± 0.24
Active smokers (%)	8 (20)	2 (25)	4 (18)	0 (0)
BNP (pg/ml)	924 ± 2412^a^	871 ± 1233^a^	142 ± 174^a^	n/a
NYHA class				
I (%)	11 (28)	2 (25)	8 (36)	n/a
II (%)	11 (28)	5 (63)	9 (41)	n/a
III (%)	17 (42)	1 (12)	4 (18)	n/a
IV (%)	1 (2)	0 (0)	1 (5)	n/a
Aetiology of HF				
DCM (%)	19 (48)	2 (25)	2 (9)	n/a
HCM (%)	3 (8)	0 (0)	4 (18)	n/a
ICM (%)	10 (25)	1 (13)	4 (18)	n/a
Inflammatory (%)	1 (2)	2 (25)	4 (18)	n/a
Other (non-ICMP) (%)	7 (17)	3 (37)	8 (36)	n/a
CMR				
LVEDVi (ml/m^2^ ± SD)	136.1 ± 49.7	117.6 ± 18.6	87.4 ± 21.2	76.6 ± 11.0
LVESVi (ml/m^2^ ± SD)	103.3 ± 47.4	69.8 ± 20.0	35.0 ± 11.3	29.3 ± 6.4
LVMi (g/m^2^ ± SD)	76.0 ± 24.1	83.6 ± 23.3	62.3 ± 24.5	57.2 ± 8.9
LVCI (ml/min/m^2^ ± SD)	2.5 ± 1.0	3.5 ± 1.1	3.9 ± 1.3	3.1 ± 0.7
LVEF (% ± SD)	25 ± 8.7	43.3 ± 2.6	60.1 ± 8.1	61.6 ± 5.6
RVEDVi (ml/m^2^)	86.5 ± 46.1^a^	104.9 ± 11.2^a^	85.8 ± 11.5^a^	87.5 ± 15.5^a^
RVESVi (ml/m^2^)	63.9 ± 31.4^a^	70.8 ± 33.3^a^	49.8 ± 5.2^a^	44.7 ± 12.6^a^
RVCI (ml/min/m^2^ ± SD)	1.9 ± 1.0	2.1 ± 0.8	2.6 ± 1.0	2.8 ± 0.7
RVEF (%)	26.5 ± 11.7^a^	32.1 ± 10.1^a^	42.3 ± 8.1^a^	49.3 ± 8.4
Native T1 (ms ± SD)	1290 ± 57^a^	1300 ± 55	1256 ± 82	1188 ± 46
ECV (% ± SD)	31.5 ± 6.3	32.6 ± 10.1	27.7 ± 5.0	24.3 ± 2.2
Scar burden (% ± SD)	6.5 ± 6.5	5.9 ± 14.6^a^	6.7 ± 6.6	0.0 ± 0.0
RV/LV T2 ratio (± SD)	0.50 ± 0.11	0.50 ± 0.10	0.54 ± 0.14	0.74 ± 0.05

*HFrEF* heart failure with reduced ejection fraction, *HFmrEF* heart failure with mildly reduced ejection fraction, *HFpEF* heart failure with preserved ejection fraction, *BMI* body mass index, *BSA* body surface area, *BNP* brain natriuretic peptide, *NYHA* New York Heart Association, *HF* heart failure, *DCM* dilated cardiomyopathy, *HCM* hypertrophic cardiomyopathy, *ICMP* ischemic cardiomyopathy, *CMR* cardiac magnetic resonance imaging, *LV* left ventricular, *EDVi* end-diastolic volume index, *ESVi* end-systolic volume index, *CI* cardiac output index, *EF* ejection fraction, *LVMi* left ventricular mass index, *RV* right ventriclular, *ECV* extracellular volume fraction

^a^Median ± interquartile range

### 6-min walking test

All HF patients underwent 6MWT while HC did not. The mean distance reached by the HF patients was 425 ± 132 m and 19 (27%) patients rated their post-exercise dyspnoea as 4 (“somewhat severe”) or higher on the modified Borg dyspnoea scale. There were no significant differences in baseline and post-exercise RR_sys_, HR or SpO_2_ between patients with and without relevant post-exercise dyspnoea. However, patients with dyspnoea reached significantly lower distances (314 ± 134 m vs. 466 ± 105 m, p < 0.001, Table 3).

**Table 3:** Baseline and post-exercise physiology

	Dyspnoea (n = 19)	No dyspnoea (n = 51)	p-value
LVEDVi (ml/m^2^ ± SD)	121.4 ± 55.0	117.7 ± 41.7	0.765
LVESVi (ml/m^2^ ± SD)	84.6 ± 56.2	68.3 ± 58.2^a^	0.658^b^
LVMi (g/m^2^ ± SD)	74.5 ± 24.4	70.0 ± 26.6^a^	0.488^b^
LVCI (ml/min/m^2^ ± SD)	2.8 ± 1.3	3.1 ± 1.3	0.319
LVEF (% ± SD)	35.9 ± 20.0	39.0 ± 17.2	0.531
RVEDVi (ml/m^2^ ± SD)	91.7 ± 40.7	89.0 ± 31.5^a^	0.300^b^
RVESVi (ml/m^2^ ± SD)	66.9 ± 36.8	55.5 ± 26.2^a^	0.879^b^
RVCI (ml/min/m^2^ ± SD)	1.9 ± 1.0	2.2 ± 1.1	0.223
RVEF (% ± SD)	27.8 ± 13.0	33.3 ± 13.3	0.130
Native T1 (ms ± SD)	1310 ± 84	1272 ± 64	0.105
ECV (% ± SD)	33.3 ± 8.6	29.1 ± 5.1	0.064
Scar burden (% ± IQR)	8.8 ± 5.0	4.2 ± 8.3^a^	**0.011**
BNP (pg/ml)	1394 ± 3410^a^	403 ± 697^a^	0.225
RV/LV T2 ratio (± SD)	0.43 ± 0.11	0.54 ± 0.11	**< 0.001**
RR_sys_ (pre; mmHg ± SD)	120.5 ± 20.9	122.4 ± 22.2	0.757
RR_sys_ (post; mmHg ± SD)	137.1 ± 23.4	136.6 ± 22.9	0.931
HR (pre; 1/min ± SD)	79 ± 15	72 ± 14	0.075
HR (post; 1/min ± SD)	98 ± 18	93 ± 18	0.281
SpO_2_ (pre; % ± SD)	97 ± 2	97 ± 1	0.226
SpO_2_ (post; % ± SD)	97 ± 2	97 ± 2	0.799
Distance (m ± SD)	314 ± 134	466 ± 105	**< 0.001**

Post-exercise dyspnoea was subjectively judged based on a modified Borg dyspnoea scale and values ≥ 4 (“somewhat severe”) were considered significant. Significant differences are highlighted in bold

*LV* left ventricular, *EDVi* end-diastolic volume index, *ESVi* end-systolic volume index, *CI* cardiac output index, *EF* ejection fraction, *LVMi* left ventricular mass index, *RV* right ventriclular, *ECV* extracellular volume fraction, *BNP* brain natriuretic peptide, *RR*_*sys*_ systolic blood pressure, *HR* heart rate, *SpO*_*2*_ peripheral capillary oxygen saturation

^a^Median ± interquartile range

^b^Derived from Mann–Whitney U test

### Reproducibility analysis

There was excellent agreement between the RV/LV T2 ratio derived from the circular ROIs compared to planar ROIs encompassing the entire ventricular cavity (r^2^ = 0.89, mean difference 0.0008 ± 0.040, limits of agreement − 0.077, 0.077). High rates of intrarater (ICC ≥ 0.93) as well as interrater reproducibility (ICC ≥ 0.96) were found for both methods. In addition, strong intraclass correlations were observed for measurements derived from basal, midventricular and apical short axis slices and four-chamber view long axis slices (all ICC ≥ 0.88).

### Relationship between CMR and 6-min walking test

**Fig. 3:**
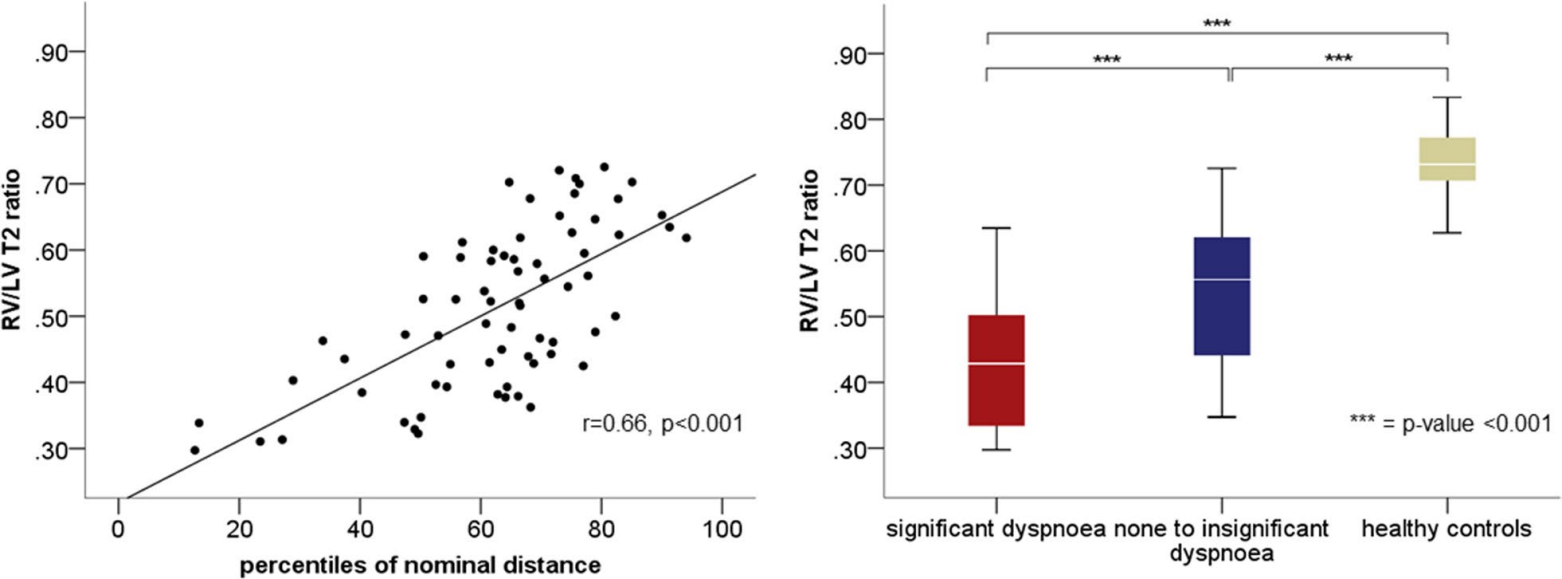
Correlation of the RV/LV T2 ratio with the percentiles of nominal distance (left) and boxplots showing the relationship of the RV/LV T2 ratio with post-exercise dyspnea (right). Brackets with asterisks mark significant differences. *RV* right ventricle, *LV* left ventricle

Median time between CMR and 6MWT was 62 ± 60 days. There was a moderate correlation (r = 0.66, p < 0.001) between the RV/LV T2 ratio and the percentile of the adjusted nominal distances (Fig. 3, left panel). Neither biventricular functional parameters nor T1 mapping showed significant correlations with the RV/LV T2 ratio (all r < ± 0.23, p > 0.05) while the scar burden showed a weak inverse correlation (r = − 0.29, p = 0.014). In addition, there were significant differences in the RV/LV T2 ratio between patients with relevant post-exercise dyspnoea, none-to-insignificant dyspnoea and HC (0.43 ± 0.11 vs. 0.54 ± 0.11 vs. 0.74 ± 0.05, all p < 0.001) (Fig. 3, right panel). There were no significant differences in LV volume indices or EF between patients with relevant post-exercise dyspnoea and those without.

In addition, there was a clear trend towards lower RV/LV T2 ratios in patients with higher NYHA classes with significant differences between all classes (0.60 ± 0.10 vs. 0.51 ± 0.10 vs. 0.44 ± 0.11 vs. 0.38 ± 0.12, NYHA I–IV respectively) and HC (0.74 ± 0.05, all p < 0.001) as well as NYHA I versus other classes (p ≤ 0.012). There were no significant differences amongst patients with HFrEF (0.50 ± 0.11), HFmrEF (0.50 ± 0.10) and HFpEF (0.54 ± 0.14). Detailed results from the post-hoc subgroup analyses between types of HF and HC can be found in Additional file 1: Table S2.

**Fig. 4:**
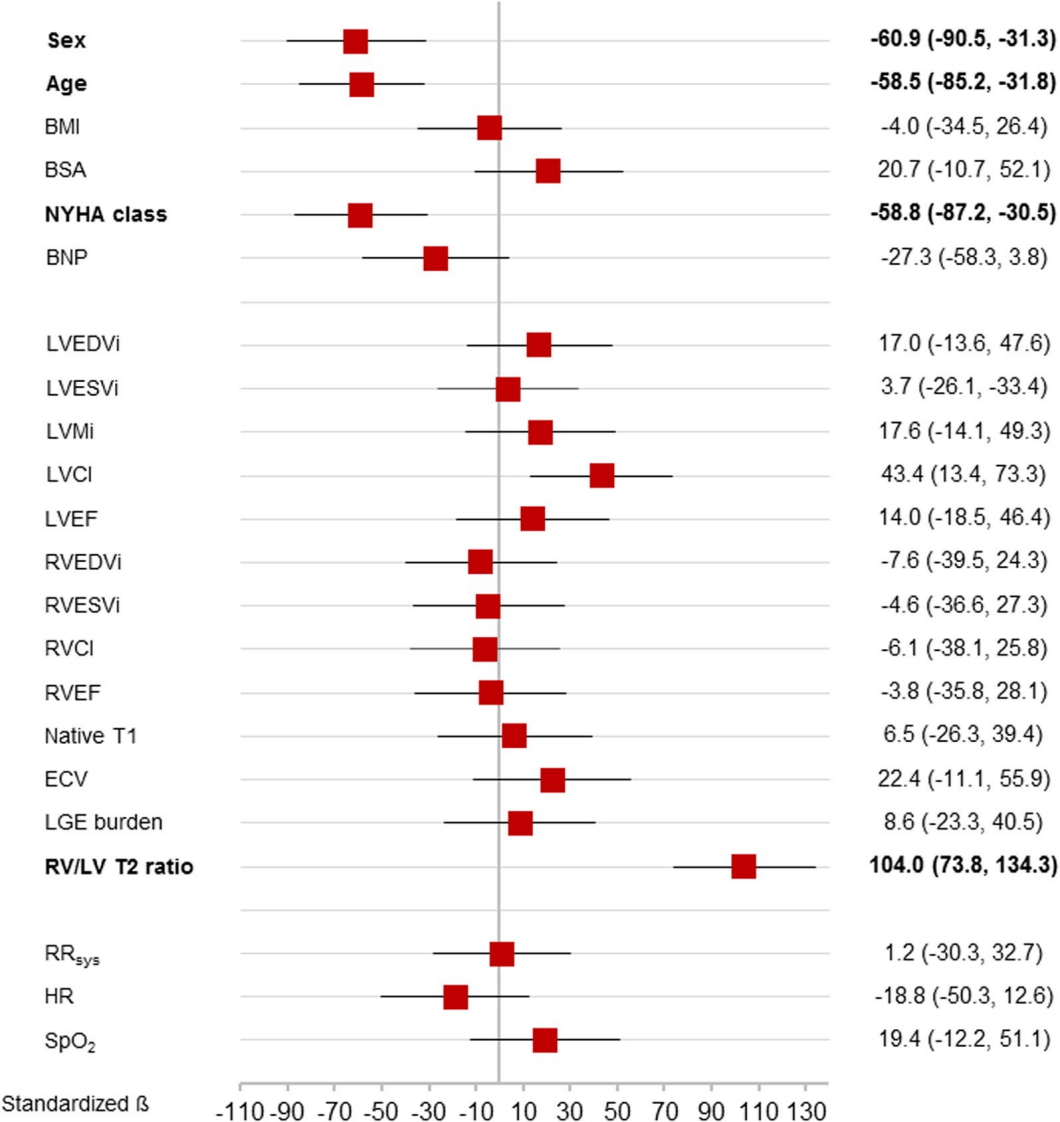
Forest plots for the hazard ratios derived from univariate regression analyses for the prediction of the distance reached in the 6MWT. Significant predictors are highlighted in bold. *BMI* body mass index, *BSA* body surface area, *NYHA* New York Heart Association, *EDVi* end-diastolic volume index, *ESVi* end-systolic volume index, *CI* cardiac output index, *EF* ejection fraction, *RV* right ventricular, *LV* left ventricular, *RR*_*sys*_ systolic blood pressure, *SpO*_*2*_ peripheral capillary oxygen saturation

Linear regression analyses showed that the RV/LV T2 ratio was the strongest tested predictor for the distance reached in the 6MWT among sex, age, NYHA class and CI (Fig. 4). Subsequent multivariate regression analysis including those five variables revealed that, among sex and age, the RV/LV T2 ratio remained an independent predictor for the distance reached in the 6MWT (Table 4). In the small subset of 10 (14%) patients, which had objective lung function analysis data available, univariate regression analysis revealed that pulmonary vital capacity, forced expiratory volume in 1 s, peak expiratory flow and pulmonary resistance were significant predictors of the distance walked in 6MWT. However, due to the small sample size, multivariate analysis was not feasible. Further, univariate logistic regression analyses revealed that the RV/LV T2 ratio was the only predictor for significant post-exercise dyspnoea (ß = − 0.23 [− 0.35, − 0.10], p = 0.001).

**Table 4:** Regression analyses for distance walked in 6MWT

Variable	Univariate hazard ratio	p-value	Multivariate hazard ratio	p-value
Demographics				
Sex	− 60.9 (− 90.5, − 31.3)	**< 0.001**	− 49.4 (− 69.8, − 29.0)	**< 0.001**
Age	− 58.5 (− 85.2, − 31.8)	**< 0.001**	− 29.7 (− 48.8, − 10.6)	**0.003**
BMI	− 4.0 (− 34.5, 26.4)	0.792	…	…
BSA	20.7 (− 10.7, 52.1)	0.193	…	…
Smoker status	20.2 (− 8.0, 48.4)	0.157	…	…
NYHA class	− 58.8 (− 87.2, − 30.5)	**< 0.001**	− 0.5 (− 29.5, 17.3)	0.603
BNP	− 27.3 (− 58.3, 3.8)	0.084	…	…
CMR				
LVEDVi (ml/m^2^)	17.0 (− 13.6, 47.6)	0.271	…	…
LVESVi (ml/m^2^)	3.7 (− 26.1, 33.4)	0.804	…	…
LVMi (g/m^2^)	17.6 (− 14.1, 49.3)	0.272	…	…
LVCI (ml/min/m^2^)	43.4 (13.4, 73.3)	**0.005**	15.8 (− 4.3, 36.0)	0.121
LVEF (%)	14.0 (− 18.5, 46.4)	0.394	…	…
RVEDVi (ml/m^2^)	− 7.6 (− 39.5, 24.3)	0.637	…	…
RVESVi (ml/m^2^)	− 4.6 (− 36.6, 27.3)	0.773	…	…
RVCI (ml/min/m^2^)	− 6.1 (− 38.1, 25.8)	0.702	…	…
RVEF (%)	− 3.8 (− 35.8, 28.1)	0.811	…	…
Native T1 (ms)	6.5 (− 26.3, 39.4)	0.693	…	…
ECV (%)	22.4 (− 11.1, 55.9)	0.186	…	…
Scar burden (%)	8.6 (− 23.3, 40.5)	0.594	…	…
RV/LV T2 ratio	104.0 (73.8, 134.3)	**< 0.001**	84.8 (56.2, 113.4)	**< 0.001**
Baseline physiology				
RR_sys_ (mmHg)	1.2 (− 30.3, 32.7)	0.940	…	…
HR (min^−1^)	− 18.8 (− 50.3, 12.6)	0.235	…	…
SpO_2_ (%)	19.4 (− 12.2, 51.1)	0.225	…	…

*6MWT* 6-min walk test, *BMI* body mass index, *BSA* body surface area, *NYHA* New York Heart Association, *BNP* brain natriuretic peptide, *CMR* cardiac magnetic resonance imaging, *LV* left ventricular, *EDVi* end-diastolic volume index, *ESVi* end-systolic volume index, *CI* cardiac output index, *EF* ejection fraction, *RV* right ventricular, *ECV* extracellular volume fraction, *RR*_*sys*_ systolic blood pressure, *SpO*_*2*_ peripheral capillary oxygen saturation

Significant predictors are highlighted in bold

## Discussion

This study investigated the relationship between RV-to-LV blood pool T2 relaxation times and exercise capacity in HF patients. The two main findings of this study can be summarized as follows: First, the RV/LV T2 ratio showed a moderate correlation with the distance patients with HF reached in the 6MWT. Second, age, sex and the RV/LV T2 ratio independently predicted the distance walked while the RV/LV T2 ratio was the only predictor of post-exercise dyspnoea.

HF is a major global health problem with increasing relevance in aging populations. All major cardiovascular societies have published HF guidelines with consistent recommendations for the diagnosis, treatment and risk stratification. In these, CMR has a central role for the analysis of ventricular function and especially for tissue characterization [12, 25].

However, routine CMR only evaluates cardiac function at rest in a supine position, in which most patients with heart failure in NYHA stage lower than 3 are asymptomatic. This could also explain why, in agreement with previous literature, no significant correlations between the 6MWT and parameters of LV function at rest were found [9, 26, 27].

This diagnostic discrepancy between rest cardiac volumetry and symptom burden is especially relevant in patients whose day-to-day activity level approaches the maximal exercise capacity and in patients with preserved ejection fraction (HFpEF). In such patients, additional exercise test imaging has been shown to substantially improve the detection of HF [28–30], and therefore is adopted into current HF guidelines [31]. However, propositions such as treadmill exercise with subsequent CMR at maximum stress yield logistical difficulties in clinical reality and are therefore not routinely employed. In-scanner exercise using ergometers and real-time CMR sequences have been shown to accurately quantify volumes at rest and stress but require dedicated equipment, training of personnel, longer scan times and extensive post-processing capabilities [32–34]. The proposed RV/LV T2 ratio, on the other hand, utilizes data, which can be easily acquired during a routine scan. In addition, there is no need for complex post-processing capabilities, as ROIs can be placed in the ventricular blood pool either in-line on the scanner or afterwards using a simple digital imaging and communications in medicine (DICOM) viewer.

Oxygen take-up in the lungs and delivery to the tissues rely on haemoglobin. The relationship between oxygen and haemoglobin is commonly described through the oxygen dissociation curve. In brief, physiological compensation mechanisms either increase oxygen affinity to improve oxygen uptake (left shift) or decrease it to facilitate delivery to target tissues (right shift) [35, 36]. It is important to note that these shifts reciprocally impact each other. According to Fick’s law, another important factor for the efficiency of gaseous exchange is the surface available at the site of diffusion.

In HF patients, upregulation of the exchange surface (i.e. increased perfusion of the capillary beds) is limited and the oxygen dissociation curve therefore has to shift to the right. Hence, it can be hypothesized that blood oxygenation levels at the end of the systemic circulation (i.e. in the RV) are lower due to increased oxygen delivery at the target tissues.

T2 mapping usually plays an important role for non-invasive tissue characterization [13]. In heart failure patients, it is used to detect myocardial oedema and chronic inflammation, especially in patients with dilated cardiomyopathy [12, 37, 38]. In this study, however, the known correlation of T2 relaxation times with blood oxygenation levels was used to investigate right-to-left ventricular differences in HF patients. Despite not directly quantifying oxygen saturation, the feasibility of this approach as a screening tool for right-to-left ventricular shunts in patients with enlarged RV has already been demonstrated [17].

While T2-based non-invasive methods for direct quantification of blood oxygenation levels exist, they mostly require scanner-specific calibration, time-consuming examination protocols, or extensive post-processing [15, 39–42]. For instance, Varghese et al. proposed a method requiring the acquisition of four separate T2 maps, which were then fed into a Luz-Meiboom model for the estimation of oxygen saturation [41].

Despite not allowing direct quantification, the mean RV/LV T2 ratio was significantly different between HF patients exhibiting relevant post-exercise dyspnoea, none-to-insignificant dyspnoea and HC. Thus, additional physiological information, which could not be extracted from established parameters such as biventricular function or myocardial tissue characterization, was derived from a routine resting-state CMR. Furthermore, the RV/LV T2 ratio showed a stronger correlation to reduced exercise capabilities of patients with heart failure than the relative myocardial scar burden while biventricular volumetric parameters showed no significant association with exercise capacities at all.

As this is study had a cross-sectional design, causal relationship between the RV/LV T2 ratio and reduced exercise capacity will have to be determined in further studies. Similarly, prognostic implications of the reported data will have to be subject to further studies, despite the fact that the 6MWT is already an established independent predictor for prognosis. In a clinical setting, this could then lead to the RV/LV T2 ratio potentially being used to identify patients needing a closer follow-up and be evaluated as an easy-to-use tool to monitor therapeutic success.

Another interesting approach would be to apply the proposed method to exercise CMR in order to compare rest and exercise RV/LV T2 ratios, potentially making exercise reserve quantifiable by CMR.

### Limitations

This is a single centre retrospective study performed with a single type of commercially available T2 mapping sequence. Despite this approach not needing a specifically tailored pulse sequence, results have to be validated at different field strengths and using different sequences. To facilitate this, a RV/LV ratio was chosen rather than RV T2 blood pool relaxation times alone. In previous work, the ratio approach has demonstrated convincing agreement at field strengths of 1.5 and 3 T [17]. In addition, the placement of the ROIs has to be performed very carefully and under consideration of the source data of the maps in order to avoid flow artefacts. In some cases, severe artefacts may limit the use of this technique in clinical practice and potential applications in exercise CMR. While feasibility of T1 mapping in exercise CMR has been shown, further studies will have to confirm the findings for T2 mapping before the proposed rest-to-stress RV/LV T2 ratio can be evaluated further. Third, the proposed RV/LV T2 ratio does not allow direct quantification of blood oxygenation and was not invasively verified at the time of CMR. In addition, during the time between CMR and 6MWT medical therapy could have influenced the results of the 6MWT and therefore biased the results. However, at the time of 6MWT none of the patients had a higher NYHA class than at time of CMR and no patients decompensated between examinations.

While this study demonstrated that the RV/LV T2 ratio is an independent predictor of exercise capacity in patients with HF, further studies will have to evaluate whether it is also an independent predictor of prognosis in these patients. Similarly, it is important to recognize pulmonary function as an additional factor on exercise function. In this retrospective study design, patients with known pulmonary disease as the leading cause of symptoms were therefore excluded. Nevertheless, in the small subset of patients with objective lung function data, it was shown that reduced pulmonary function is a significant predictor for less distance walked in the 6MWT. Unfortunately, the subgroup was too small for multivariate regression analysis and therefore, future prospective validation efforts will have to evaluate the interrelationship between the RV/LV T2 ratio and pulmonary function.

Furthermore, this study included patients with HF due to all aetiologies and also investigated patients with HFrEF, HFmrEF as well as HFpEF, resulting in a heterogeneous cohort of patients. Because of the size of the HFmrEF subgroup (n = 8) and due to the fact that it only included males, results of the post-hoc analysis have to be interpreted cautiously as they might not be representative for this cohort. Further studies will have to investigate whether results are applicable for sub-cohorts (i.e. HFpEF only). Finally, multiple equations for the calculation of age-, height-, weight-, and gender-adjusted nominal distances from 6MWT have been proposed and validated. The most commonly-used equations are by Troosters et al. [23] and Enright et al. [43]. This study is based on the former formula, however, as there is an almost perfect correlation between nominal distances of the two (r = 0.97 for our data), the results are considered transferable.

## Conclusion

The proposed RV/LV T2 ratio is a simple tool using routinely acquired data in the diagnostic work-up of HF patients. It independently predicts exercise capabilities and moderately correlates with the 6MWT. Further studies are needed to investigate its relationship to prognosis and its potential to aid in selecting patients who need a closer follow-up.

## Supplementary Information

**Additional file 1: Table S1.** Comparison of CMR parameters between patient groups. **Table S2.** Results from post-hoc tests between types of HF and HC. **Table S3.** Multivariate regression analyses for distance walked in 6MWT. **Table S4.** Univariate regression analyses for distance walked in 6MWT.

## Data Availability

The datasets analysed during the current study available from the corresponding author on reasonable request.

## Abbreviations

6MWT: 6-Minute walk test
ANOVA: Univariate analysis of variance
BMI: Body mass index
BNP: Brain natriuretic peptide
BSA: Body surface area
bSSFP: Balanced steady-state free precession
CI: Cardiac output index
CMR: Cardiac magnetic resonance imaging
EACVI: European Association for Cardiovascular Imaging
ECG: Electrocardiogram
ECV: Extracellular volume fraction
EDV: End-diastolic volume
EF: Ejection fraction
ESC: European Society of Cardiology
ESV: End-systolic volume
HC: Healthy controls
HF: Heart failure
HFmrEF: Heart failure with mildly reduced ejection fraction
HFpEF: Heart failure with preserved ejection fraction
HFrEF: Heart failure with reduced ejection fraction
HR: Heart rate
LGE: Late gadolinium enhancement
LV: Left ventricle
MACE: Major cardiac adverse events
MRI: Magnetic resonance imaging
NYHA: New York Heart Association
ROI: Region of interest
RR_sys_: Systolic blood pressure
RV: Right ventricle
SCMR: Society for Cardiovascular Magnetic Resonance
SpO_2_: Peripheral capillary oxygen saturation
T: Tesla
