# Supplementary material for: Cardiac MRI-based right-to-left ventricular blood pool T2 relaxation times ratio correlates with exercise capacity in patients with chronic heart failure
Source: J Cardiovasc Magn Reson. 2023 Jun 19;25:33. doi: 10.1186/s12968-023-00943-y (PMC10278263; doi:10.1186/s12968-023-00943-y)
Supplement: Supplementary file 1 — Additional file 1: Table S1. Comparison of CMR parameters between patient groups. Table S2. Results from post-hoc tests between types of HF and HC. Table S3. Multivariate regression analyses for distance walked in 6MWT. Table S4. Univariate regression analyses for distance walked in 6MWT. [file 12968_2023_943_MOESM1_ESM.docx]

| **Table S1** Comparison of CMR parameters between patient groups | | | |
| --- | --- | --- | --- |
|  | acute indication for CMR (n=28) | scheduled follow-up CMR (n=42) | p-value |
| LVEDVi (ml/m² ± SD) | 126.3 ± 47.7 | 113.6 ± 43.5 | 0.251 |
| LVESVi (ml/m² ± SD) | 88.1 ± 50.8 | 71.3 ± 45.7 | 0.156 |
| LVMi (g/m^2^ ± SD) | 72.9 ± 17.1 | 72.3 ± 29.2 | 0.920 |
| LVCI (ml/min/m² ± SD) | 3.0 ± 1.3 | 3.1 ± 1.3 | 0.894 |
| LVEF (%± SD) | 33.8 ± 15.6 | 41.0 ± 18.9 | 0.100 |
| RVEDVi (ml/m² ± SD) | 92.3 ± 24.8 | 94.1 ± 34.4 | 0.809 |
| RVESVi (ml/m² ± SD) | 64.3 ± 22.8 | 64 ± 30.4 | 0.967 |
| RVCI (ml/min/m² ± SD) | 1.9 ± 1.0 | 2.2 ± 1.0 | 0.280 |
| RVEF (% ± SD) | 30.4 ± 13.3 | 32.7 ± 13.5 | 0.493 |
| Native T1 (ms ± SD) | 1288 ± 81 | 1279 ± 67 | 0.649 |
| ECV (% ± SD) | 29.3 ± 6.0 | 31.0 ± 6.9 | 0.292 |
| Scar burden (%± SD) | 6.4 ± 5.3 | 6.7 ± 6.3 | 0.794 |
| RV/LV T2 ratio (± SD) | 0.50 ± 0.12 | 0.52 ± 0.12 | 0.357 |
| CMR, cardiac magnetic resonance imaging; LV, left ventricular; EDVi, end-diastolic volume index; ESVi, end-systolic volume index; CI, cardiac output index; EF, ejection fraction; LVMi, Left ventricular mass index; RV, right ventriclular; ECV, extracellular volume fraction; LGE, late gadolinium enhancement | | | |

| **Table S2** Results from post-hoc tests between types of HF and HC | | | | | | | | | | | | |
| --- | --- | --- | --- | --- | --- | --- | --- | --- | --- | --- | --- | --- |
|  | HFrEF vs. HFmrEF | | HFrEF vs. HFpEF | | HFmrEF vs. HFpEF | | HFrEF vs. HC | | HFmrEF vs. HC | | HFpEF vs.HC | |
|  | Diff.* | p-value | Diff. | p-value | Diff. | p-value | Diff. | p-value | Diff. | p-value | Diff. | p-value |
| BMI (kg/m²) | 3.6 | 0.446 | -2.7 | 0.332 | **-6.3** | **0.024** | 2.4 | 0.306 | -1.2 | 1.000 | **5.1** | **0.003** |
| BSA (m²) | 0.05 | 1.000 | -0.09 | 0.989 | -0.14 | 0.952 | 0.04 | 1.000 | -0.01 | 1.000 | 0.13 | 0.270 |
| BNP (pg/ml) | 53 | 1.000 | 782 | 0.369 | 729 | 1.000 | n/a | n/a | n/a | n/a | n/a | n/a |
| LVEDVi (ml/m²) | 18.4 | 0.942 | **48.6** | **<0.001** | 30.2 | 0.183 | **59.4** | **<0.001** | **41.0** | **0.013** | 10.8 | 1.000 |
| LVESVi (ml/m²) | **33.5** | **0.034** | **68.4** | **<0.001** | **34.8** | **0.042** | **74.0** | **<0.001** | **40.5** | **0.006** | 5.7 | 1.000 |
| LVMi (g/m^2^) | -7.6 | 1.000 | 13.6 | 0.079 | 21.3 | 0.078 | **18.8** | **0.001** | **26.4** | **0.008** | 5.1 | 1.000 |
| LVCI (ml/min/m²) | **-1.0** | **0.046** | **-1.5** | **<0.001** | -0.4 | 1.000 | -0.6 | 0.079 | 0.5 | 1.000 | **0.9** | **0.008** |
| LVEF (%) | **-18.3** | **<0.001** | **-35.1** | **<0.001** | **-16.8** | **<0.001** | **-36.6** | **<0.001** | **-18.3** | **<0.001** | -1.5 | 1.000 |
| RVEDVi (ml/m²) | -22.7^ꝉ^ | 0.089^ꝉ^ | -1.7^ꝉ^ | 0.988^ꝉ^ | 19.1 | 0.505 | -1.5^ꝉ^ | 0.857^ꝉ^ | 17.4 | 0.576 | -1.6 | 1.000 |
| RVESVi (ml/m²) | -11.5^ꝉ^ | 0.223^ꝉ^ | **14.2^ꝉ^** | **0.018^ꝉ^** | **25.7^ꝉ^** | **<0.001^ꝉ^** | **19.3^ꝉ^** | **<0.001^ꝉ^** | **26.0** | **0.023** | 5.1^ꝉ^ | 0.057 |
| RVCI (ml/min/m²) | -0.3 | 1.000 | **-0.7** | **0.028** | -0.4 | 1.000 | **-0.9** | **<0.001** | -0.6 | 0.492 | -0.2 | 1.000 |
| RVEF (%) | -5.6 | 1.000 | **-15.6^ꝉ^** | **<0.001^ꝉ^** | 9.0**^ꝉ^** | 0.050**^ꝉ^** | **-22.8** | **<0.001** | **-17.2** | **0.001** | **-8.2**^ꝉ^ | **0.008^ꝉ^** |
| Native T1 (ms) | -31^ꝉ^ | 0.653^ꝉ^ | **59^ꝉ^** | **0.032^ꝉ^** | 43 | 0.707 | **92^ꝉ^** | **<0.001^ꝉ^** | **112** | **<0.001** | **68** | **0.001** |
| ECV (%) | -1.1 | 1.000 | 3.8 | 0.057 | 4.9 | 0.207 | **7.2** | **<0.001** | **8.3** | **0.001** | 3.4 | 0.116 |
| Scar burden (%) | -0.1 | 1.000 | -0.3 | 1.000 | -0.2 | 1.000 | **6.5** | **<0.001** | **5.9** | **<0.001** | **6.7** | **<0.001** |
| RV/LV T2 ratio | 0.00 | 1.000 | -0.04 | 1.000 | -0.04 | 1.000 | **-0.24** | **<0.001** | **-0.24** | **<0.001** | **-0.20** | **<0.001** |
| HF, heart failure; HC, healthy controls; HFrEF, heart failure with reduced ejection fraction; HFmrEF, heart failure with mildly reduced ejection fraction; HFpEF, heart failure with preserved ejection fraction; BMI, body mass index; BSA, body surface area; BNP, brain natriuretic peptide; LV, left ventricular; EDVi, end-diastolic volume index; ESVi, end-systolic volume index; CI, cardiac output index; EF, ejection fraction; LVMi, Left ventricular mass index; RV, right ventriclular; ECV, extracellular volume fraction; LGE, late gadolinium enhancement  *mean difference between respective groups  ^ꝉ^difference of Medians, p-value derived from Mann-Whitney U Test  Statistically significant differences are bolded. | | | | | | | | | | | | |

| **Table S3** Multivariate regression analyses for distance walked in 6MWT | | | |
| --- | --- | --- | --- |
| Variable | Multivariate Hazard Ratio | p-value | Variance inflation factor |
| Demographics |  |  |  |
| Sex | -49.4 (-69.8, -29.0) | **<0.001** | 1.07 |
| Age | -29.7 (-48.8, -10.6) | **0.003** | 1.13 |
| NYHA class | -0.5 (-29.5, 17.3) | 0.603 | 1.48 |
| CMR |  |  |  |
| LVCI | 15.8 (-4.3, 36.0) | 0.121 | 1.13 |
| RV/LV T2 ratio | 84.8 (56.2, 113.4) | **<0.001** | 1.53 |
| 6MWT, 6-minute walk test; NYHA, New York Heart Association; LV, left ventricular; CI, cardiac output index; RV, right ventricular;  Independently significant predictors are highlighted in bold. | | | |

| **Table S4** Univariate regression analyses for distance walked in 6MWT | | |
| --- | --- | --- |
| Pulmonary function (% of normal) | Univariate Hazard Ratio | p-value |
| Total lung capacity | 58.8 (-49.8, 167.4) | 0.315 |
| Vital capacity | -105.0 (-182.3, -27.2) | **0.027** |
| Residual volume | -55.7 (-165.1, 53.7) | 0.342 |
| FEV1 | -114.6 (-185.2, -44.0) | **0.012** |
| PEF | 133.5 (73.7, 193.3) | **0.003** |
| Resistance | 119.3 (44.3, 194.3) | **0.015** |
| 6MWT, 6-minute walk test; FEV1, forced expiratory volume in 1 second; PEF, peak expiratory flow | | |
